# Efficient computational modeling of electronic stopping power of organic polymers for proton therapy optimization

**DOI:** 10.1038/s41598-024-60651-0

**Published:** 2024-04-29

**Authors:** F. Matias, T. F. Silva, N. E. Koval, J. J. N. Pereira, P. C. G. Antunes, P. T. D. Siqueira, M. H. Tabacniks, H. Yoriyaz, J. M. B. Shorto, P. L. Grande

**Affiliations:** 1 Instituto de Pesquisas Energéticas e Nucleares, Av. Professor Lineu Prestes, São Paulo, 05508-000 Brazil; 2 Instituto de Física da Universidade de São Paulo, Rua do Matão, trav. R187, São Paulo, 05508-090 Brazil; 3 Centro de Física de Materiales, Paseo Manuel de Lardizabal 5, Donostia-San Sebastián, 20018 Spain; 4 Instituto de Física da Universidade Federal do Rio Grande do Sul, Av. Bento Gonçalves, Porto Alegre, 9500 Brazil

**Keywords:** Biological physics, Atomic and molecular collision processes, Radiotherapy

## Abstract

This comprehensive study delves into the intricate interplay between protons and organic polymers, offering insights into proton therapy in cancer treatment. Focusing on the influence of the spatial electron density distribution on stopping power estimates, we employed real-time time-dependent density functional theory coupled with the Penn method. Surprisingly, the assumption of electron density homogeneity in polymers is fundamentally flawed, resulting in an overestimation of stopping power values at energies below 2 MeV. Moreover, the Bragg rule application in specific compounds exhibited significant deviations from experimental data around the stopping maximum, challenging established norms.

## Introduction

In the last two decades, clinical therapy using proton beams to treat cancerous tumors has experienced steady growth^1–5^. Although this form of radiotherapy already counts with highly developed technology, it still retains significant challenges in terms of physical and clinical aspects^6–8^. One of these challenges is the precise accounting of relative biological effectiveness (RBE), which is the ratio between the doses required by two types of radiation to cause the same biological effect. This factor, measurable through linear energy transfer (LET) or microdosimetry, depends on how the energy is deposited on a micrometric scale^9^.

In proton therapy, relative biological effectiveness (RBE) is traditionally defined by a constant value of 1.1 (relative to X-ray dose) for all points along the beam path and all stopping points^10,11^. However, a comprehensive review of the available experimental data in the literature^12^ reveals that, despite a lack of experimental standardization and large uncertainties, there is evidence that RBE values vary considerably and can exceed 1.1 at the end of the beam range. These differences have clinical implications^13,14^. Therefore, it is important to accurately reduce experimental uncertainties to describe the effects of proton beams on tissues.

When an ion with kinetic energy moves through matter, it interacts with the target electrons and nuclei, leading to deceleration. These interactions are known as electronic and nuclear stopping, respectively^15,16^. The stopping power expresses a medium’s force on the ion, leading to the mean energy loss per unit path length while traveling in that medium. In the proton-matter interaction, the electronic stopping power is dominant. There is a very small contribution from nuclear-stopping power for $v \ll v_{\text {F}}$ (with $v_{\text {F}}$ being the Fermi velocity). For the current proposal, we will focus solely on calculations of electronic stopping power.

The energy of the proton transferred to the biological tissue is directly related to its velocity. As the proton slows down, the energy transferred to the tissue per unit path length, determined by the electronic stopping power, increases, resulting in maximum dose deposition at a specific depth. This region around the peak of maximum dose deposition is known as the Bragg peak^17^ and is closely related to the stopping maximum. It is the region of greatest interest in proton beam radiotherapy applications, and its precise positioning is crucial during the definition of the irradiation plan. This particular profile of proton beam energy deposition presents significant clinical advantages, especially for pediatric patients, by allowing optimal dose delivery to tumor tissue and by minimizing dose to organs at risk in surrounding areas, thus reducing the chances of future complications and induction of secondary tumors^18–20^. On the other hand, the high and relatively narrow dose peak makes quality control in dose monitoring and precise patient positioning even more crucial, with the risk of damaging health tissues with high radiation doses. Therefore, in-depth investigations of the uncertainties in the range and stopping power values are essential for a more accurate dose distribution in patients^21,22^.

Accurate knowledge of electronic stopping power is essential in proton therapy. It is also important in many fields of science and technological applications, such as outer space exploration (space weathering), nanotechnology (ion beam modeling), material modifications, and nuclear fusion research (plasma-wall interaction)^23–27^. However, as depicted above, its most critical application lies in dosimetry for cancer treatment using ions, given the increasing global use of protons and heavier ions in radiation therapy and the risks involved^28,29^. For convenience, stopping power can be normalized by the atomic density to eliminate the dependence on the material’s density. The resulting quantity is known as the stopping cross-section (SCS). Therefore, SCS is a fundamental quantity that requires a detailed understanding of energy-loss processes.

Perturbation theories employing the dielectric function were used to compute electronic stopping power^30–36^, presenting limitations in energy ranges relevant to studying biological damage caused by the radiation. Real-time time-dependent density functional theory (TDDFT) is a non-perturbative method based on modern quantum-mechanical simulations. It has been used as an alternative approach to investigate electronic stopping in complex systems like water and DNA^37–42^. However, using the precise atomic structure of complex chemical systems, such as DNA, within real-time TDDFT requires the computation of multiple trajectories to obtain an average (random) electronic stopping power and is highly computationally demanding.

On the other side, in investigations into ion-matter interactions, it is customary to employ simplified models, exemplified by the homogeneous, free electron gas (FEG) model, to represent valence electrons within materials. This pragmatic approach facilitates straightforward predictions of stopping power and yields results that closely align with experimental data^43–49^. Although the FEG model is reliable for materials with simple electronic structures, its effectiveness diminishes when dealing with materials characterized by complex electronic excitations. Here, we demonstrate that these materials can still be treated as a collection of FEGs with high accuracy, ensuring simplicity and avoiding time-consuming full atomistic *ab initio* calculations. For this purpose, we utilized electronic stopping power for a FEG with different densities or plasmon frequencies from the real-time TDDFT calculations^43,48,50,51^. The results were averaged according to the Penn method^52^.

Knowledge of materials’ energy-loss function (ELF) is essential in this framework. The Penn approach^53^ introduced an algorithm to determine the electron inelastic mean free paths (IMFP) by utilizing a model dielectric function derived from the experimental ELF specific to the material under investigation. The same model has been applied to estimate the electron stopping power in various materials^54^ and has been extended to calculate the non-linear stopping power of ions^52^. This extension involves using the ELF to appropriately weight contributions from different electron gas components within a statistical ensemble that characterizes the material of interest. ELF functions at the optical limit can be found for different materials elsewhere^55^.

The Bragg rule has been used to calculate the stopping values for compounds such as hydrocarbons. According to this rule, the SCS per atom in a compound is the weighted average of the SCS of each of its constituent elements^17^, similar to a gas system of non-interacting atoms. The Bragg rule is considered relatively accurate for solids, with measurements of stopping powers for ions in compounds deviating less than 5% from its predictions^56^. However, the rule applicability has limitations, as the energy lost by the ions to the electrons in a material depends on its detailed orbital and excitation structure, which are affected by neighboring atoms. Additionally, these interactions can alter the charge state of the traveling ion, affecting the intensity of interactions with the medium.

According to Lodhi and Powers^57^, the Bragg rule may fail close to stopping power maximum for hydrocarbons compared to experimental data. The core and bounds (CAB) approach proposes that the stopping power of compounds can be predicted by combining the stopping caused by the atomic “core” electrons with the corresponding stopping of the bound electrons^58,59^. The core’s contribution to stopping power is determined by applying the Bragg rule to the atoms in the compound. In contrast, the bounding electrons in the compound would then include the necessary stopping correction. The CAB approach generates corrections to the Bragg rule for polymers containing light elements, such as H, C, N, and O. These light atoms have the most significant bonding effect on stopping powers. The application of these corrections is explained in reference ^59^.

By applying the proposed formalism, we aim to verify the validity of the Bragg rule and the FEG model, assuming homogeneous electron density in the context of organic polymers. Specifically, we examined the cases of polyethylene (PE), polystyrene (PS), poly(2-vinylpyridine) (P2VP), polyacetylene (PA), poly(methyl methacrylate) (PMMA) and polyimide (PI). The study of these polymers is important because virtually all phantoms used for dose verification and quality assurance in proton therapy treatments are manufactured with polymers such as PMMA. Furthermore, some components that make up the proton accelerators are constructed with PE or PS^32,60–65^.

## Results and discussion

**Figure 1:**
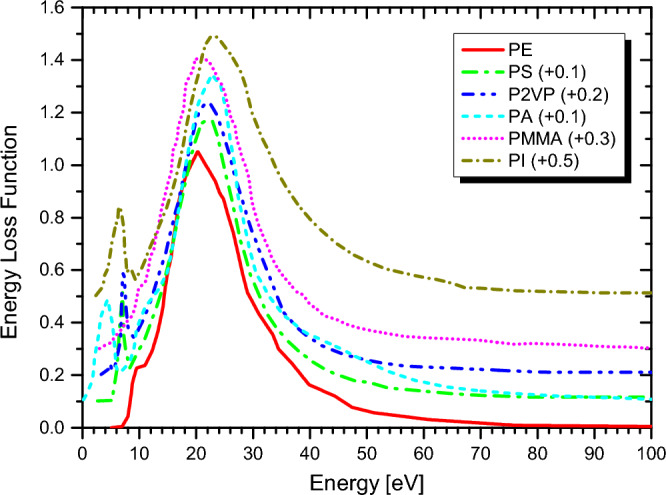
Optical-ELF data for PE, PS, P2VP, PA, PMMA, and PI obtained from^31,66,67^ and used to calculate electronic SCS with the real-time TDDFT-Penn approach.

**Figure 2:**
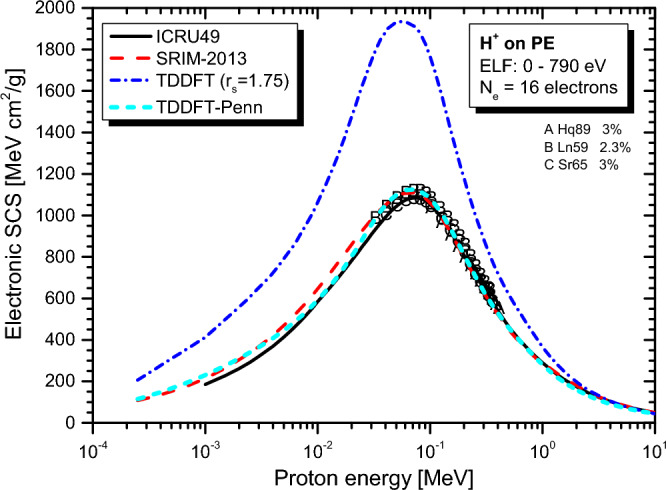
Proton SCS in PE polymer. Real-time TDDFT results with a unique FEG ($r_s=1.75$ au) are shown in the blue dash-dot line, and the real-time TDDFT-Penn is in the cyan short dash line. Experimental data (uppercase letters) around the stopping maximum^71,72^. Semi-empirical models ICRU49^68^ and SRIM-2013^70^ presented.

**Figure 3:**
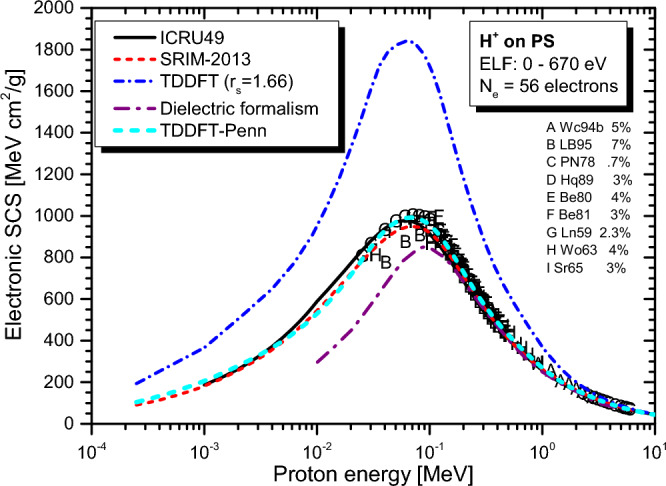
Proton SCS in PS polymer. Real-time TDDFT results using a unique FEG ($r_s=1.66$ au) and real-time TDDFT-Penn. Experimental data (uppercase letters) concentrated around the stopping maximum. Dielectric formalism results in purple dash-dot line^32^. Semi-empirical models ICRU49^68^ and SRIM-2013^70^ showcased.

**Figure 4:**
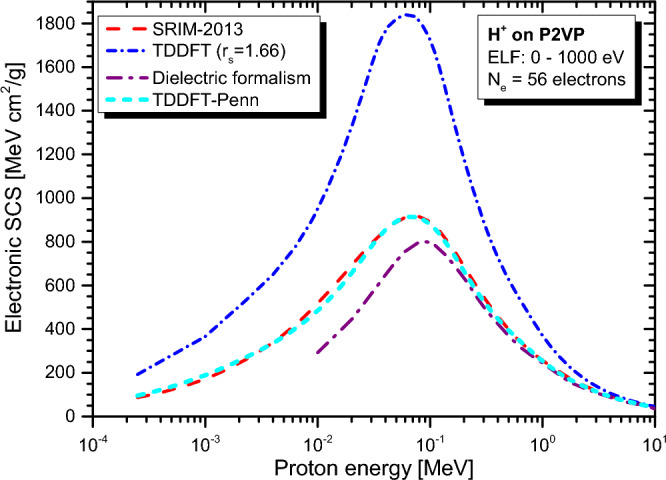
Proton SCS in P2VP polymer. Real-time TDDFT results with a unique FEG ($r_s=1.66$ au) and real-time TDDFT-Penn. Results based on dielectric formalism^31^ and the semi-empirical model SRIM-2013^70^ are also included.

**Figure 5:**
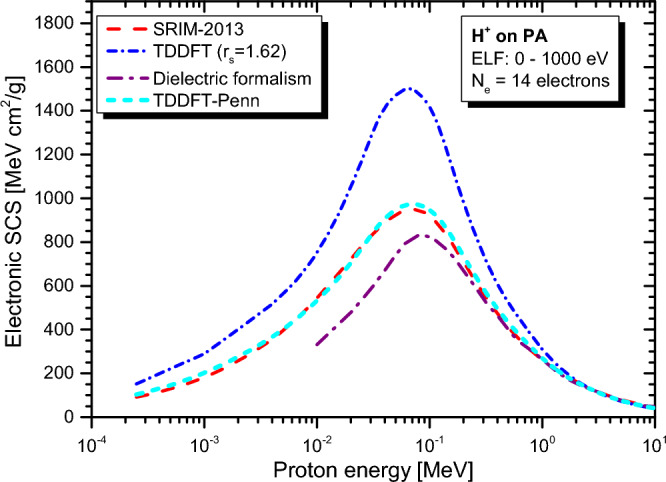
Proton SCS in PA polymer. Real-time TDDFT results with a unique FEG ($r_s=1.62$ au) and real-time TDDFT-Penn. Results based on dielectric formalism^31^. Semi-empirical model SRIM-2013^70^ presented.

**Figure 6:**
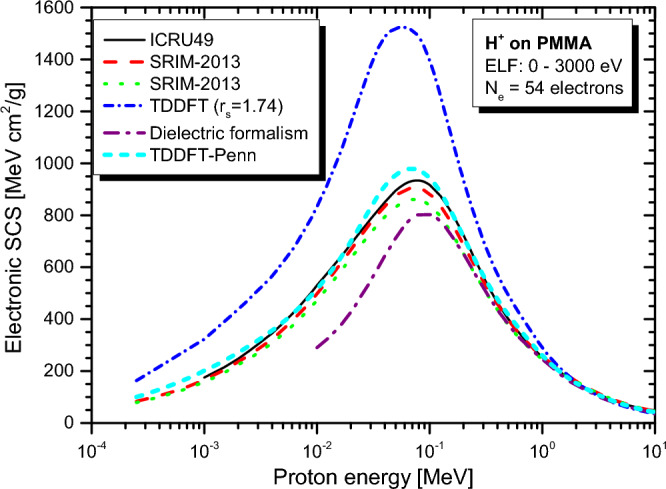
Proton SCS in PMMA polymer. Real-time TDDFT results with a unique FEG ($r_s=1.74$ au) and real-time TDDFT-Penn. Dielectric formalism results^31^. Semi-empirical models ICRU49^68^ and SRIM-2013^70^ with the Bragg rule (red dashed line) and CAB (dotted green line) correction showcased.

**Figure 7:**
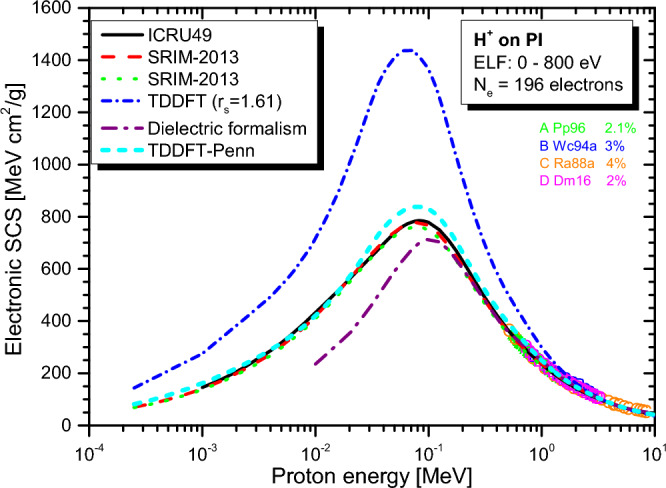
Proton SCS in PI polymer. Real-time TDDFT results with a unique FEG ($r_s=1.61$ au) and real-time TDDFT-Penn. Results based on dielectric formalism^31^. Semi-empirical models ICRU37^69^, and SRIM-2013^70^ with the Bragg rule and CAB correction presented.

Real-time TDDFT-Penn calculations were performed according to Eqs. (3) to (5), and the electronic SCS results for PE, PS, P2VP, PA, PMMA, and PI to energetic protons are presented in Fig. 2, 3, 4, 5, 6 and 7, respectively. The data used to calculate SCS with the real-time TDDFT-Penn method are listed in Table 1, and the optical-ELF data for each polymer are shown in Fig. 1.

**Table 1:** Data used in the real-time TDDFT-Penn approach to calculate the electronic SCS of different polymers based on their monomers^31,66,67^.

Polymer	Formula	ELF range (eV)	Total/valence electrons	$\rho$ (g/cm^3^)
PE	(C_2_H_4_)_n_	0–790	16/12	0.93
PS	(C_8_H_8_)_n_	0–670	56/40	1.06
P2VP	(C_7_H_7_N)_n_	0–1000	56/40	1.15
PA	(C_2_H_2_)_n_	0–1000	14/10	1.36
PMMA	(C_8_H_8_O_2_)_n_	0–3000	54/40	1.19
PI	(C_22_H_10_N_2_O_5_)_n_	0–800	196/138	1.42

We compare the results of our approach with ICRU49^68^, ICRU37^69^, SRIM-2013^70^, and real-time TDDFT with the homogeneous assumption. This comparison shows the need to completely break down the assumption of spatial homogeneity of the valence electron density in complex materials, such as polymers. For example, the homogeneous assumption leads to overestimating the SCS values for proton energies below approximately 2 MeV compared to the SRIM-2013 data. Our approach produces more realistic values and, at the same time, offers a physically sound approach to dealing with inhomogeneities.

We also included in the comparison the experimental data available at the IAEA database^71,72^ (uppercase letters), with which the real-time TDDFT-Penn results show an excellent agreement, as well as with semi-empirical SRIM-2013^70^ results (red dashed line) using the Bragg rule^17^, as can be seen in Figs. 2 and 3. SCS results from the dielectric formalism, particularly the Mermin-Energy-Loss-Function Generalized Oscillator Strength model (MELF-GOS)^31^, are also included in the comparisons. This approach also considers inhomogeneities in the material’s electron density utilizing a similar optical ELF. Thus, both methods will give similar mean excitation energies (I) (occurring in the Bethe formula for fast projectiles). Several studies have employed this approach to calculate SCS in biological media^31,32,38,73^. However, even though such a linear model typically performs better at projectile energies above the maximum for protons, it underestimates the SCS by a significant amount at the maximum for the present polymers. Because this theoretical model is linear, it loses accuracy for ion energies around the stopping maximum and below. In this energy range, the non-linear effects become significant. Even though we have not presented MELF-GOS results for PE, we expect similar behavior to the others. This issue is expected to be significantly more severe for heavier projectiles than protons.

In particular, the real-time TDDFT-Penn results for PS (refer to Fig. 3) agree better with the experimental data than SRIM-2013. SRIM-2013 employs the Bragg rule, resulting in an excitation energy (I) for PS of 65.5 eV^74^. However, electron energy loss spectroscopy (EELS) data from experiments^67^ for the compound PS suggest a lower I value (59.3 eV). This observation explains the higher values obtained with our approach around the stopping maximum.

SCS results for P2VP and PA (see Figs. 4 and 5) agree well with SRIM-2013. However, it is important to note that we did not find any experimental data for comparison in this context. Furthermore, the dielectric approach, employing a similar ELF function as input^31^, deviates significantly below the position of the stopping maximum.

Interestingly, for PMMA and PI (see Figs. 6 and 7), the Bragg rule indicates 74 eV^68^ and 79.6 eV^69^, while the experimental ELF^31^ points to significantly lower values of 66 eV and 68 eV, respectively. It is worth noting that the core and bond (CAB) correction on SRIM-2013^70^ is small but makes the deviation from our approach even higher, pointing to a correction in the opposite direction.

As shown in Figs. 2, 3, 4, 5, 6 and 7, the differences between real-time TDDFT-Penn and SRIM-2013 are small but more pronounced for PMMA and PI at the position of the stopping maximum. They can be attributed to a stronger breakdown of the Bragg rule due to the complex molecular structures of PMMA and PI, which feature bonds between C, O, and N.

Table 2 shows the different types of chemical bonds in the respective polymeric monomers of PE, PS, P2VP, PA, PMMA, and PI. PMMA and PI exhibit higher chemical structure complexity than other polymers. While PMMA has one $\hbox {C}=\hbox {O}$ and two $\hbox {C}-\hbox {O}$ bonds, PI has four $\hbox {C}=\hbox {O}$, two $\hbox {C}-\hbox {O}$ bonds, and four $\hbox {N}-\hbox {C}$ bonds. The electronegativity of the atoms in these bonds varies, with oxygen being more electronegative than nitrogen, which is more electronegative than carbon.

**Table 2:** Description of the types of bonds in the polymeric monomers PE, PS, P2VP, PA, PMMA, and PI.

Types of bonds	PE	PS	P2VP	PA	PMMA	PI
$\hbox {C}=\hbox {C}$	–	Three bonds	Two bonds	One bond	–	Nine bonds
$\hbox {C}-\hbox {C}$	One bond	Five bonds	Four bonds	–	Three bonds	Thirteen bonds
$\hbox {C}-\hbox {H}$	Four bonds	Eight bonds	Seven bonds	Two bonds	Eight bonds	Ten bonds
$\hbox {C}=\hbox {O}$	–	–	–	–	One bond	Four bonds
$\hbox {C}-\hbox {O}$	–	–	–	–	Two bonds	Two bonds
$\hbox {C}=\hbox {N}$	–	–	One bond	–	–	–
$\hbox {C}-\hbox {N}$	–	–	One bond	–	–	Four bonds

The bonds may be of type single ($\sigma$-bonds) or double ($\pi$-bonds).

Analogously, in TiN compounds, there is a transfer of 1.51 electrons from titanium to nitrogen^49^, and the transferred charges. The transfer of charges is expected to be more noticeable in the double bonds between carbon and oxygen in polymers like PMMA and PI. Therefore, our results suggest the possibility of charge transfer occurring from carbon to oxygen or nitrogen on these polymers. If this transfer occurs, employing the Bragg rule will likely lead to a decreased accuracy in predicting the SCS. Indeed, the SRIM-2013 results (red dashed line) for the PMMA and PI compounds are numerically lower in the position of the stopping maximum compared to real-time TDDFT-Penn predictions.

Finally, it should be pointed out that the consideration of neutral hydrogen (H^0^) charge states may affect the calculated stopping power around the stopping power maximum. The presence of neutral hydrogen will reduce the energy loss for target ionization and excitation but will add other energy-loss mechanisms, such as capture and electron loss. Simulations using the CasP program^75^ show that these mechanisms compensate for the reduction in ionization and excitation.

## Conclusion

Theorists have been working for decades to develop methods to model the physical processes responsible for electronic SCS. Although these methods have been successful, they have yet to be able to cover a wide range of energy using a single approach. Some models explain a limited energy range, while others achieve good agreement with experimental data by treating inner and valence electrons differently. This work presents a theoretical framework based on *ab initio* calculations of different FEGs and the Penn method. This framework achieves excellent agreement with experimental data and reference data tables across a wide range of energy using a single approach for all electrons.

To showcase our approach’s efficiency, we investigated the electronic stopping power of organic polymers for protons. Generally, a single FEG model cannot accurately describe the electronic stopping power around the stopping maximum. Our new method provides a theoretical framework to tackle electron density variations. We have demonstrated the effectiveness of our method by obtaining excellent agreement with the experimental data and the reference data tables for polymers such as PE, PS, P2VP, PA, PMMA, and PI (as shown in Figs. 2, 3, 4, 5, 6 and 7). Our findings highlight the importance of considering the intricate electronic structures of polymers in the theoretical modeling of stopping power.

These findings emphasize the importance of considering the complex electronic structures of polymers in predicting stopping power accurately. The agreement between the real-time TDDFT-Penn approach and the experimental data, indicated by uppercase letters in Figs. 2 and 3, demonstrates the theoretical framework’s precision. However, further validation is necessary, particularly for polymers with limited data.

Differences in predicted mean excitation energies and SCS magnitudes between the real-time TDDFT-Penn approach and semi-empirical models such as SRIM-2013, particularly around the stopping maximum, highlight the potential influence of molecular structure on these predictions. The distinct chemical compositions of PMMA and PI, which have varying electronegativities in their bonds, likely contribute to charge transfer effects not adequately accounted for by the Bragg rule. The primary consequence of the breakdown of the Bragg rule is a reduction in the mean excitation energy by approximately 10% and a decrease in the projectile range by about 3 mm for 200 MeV protons.

In conclusion, the results of this research challenge established assumptions and emphasize the need for precise modeling of materials with complex electronic structures. This study enhances our understanding of ion-polymer interactions. It provides a solid foundation for future applications in proton therapy and other fields where accurate predictions of stopping power are essential.

## Simulation methods

### Real-time TDDFT approach

Real-time TDDFT is a highly effective ab initio tool for describing electronic stopping power in spherical jelliums. The jellium model assumes a positive background (representing the ion cores) that provides a charge balancing for the electron gas. Compared to fully atomistic models, the advantage of such representation is computational efficiency. The real-time TDDFT in a FEG has been shown to provide accurate results for near-free-electron systems. On the other hand, using an atomistic representation requires careful consideration of trajectories to calculate the random stopping power^76,77^.

The polymeric media are modeled as jellium spheres in all the DFT and real-time TDDFT calculations. The positive background density of the jellium with radius $R_\text{cl}$ is defined by $n_{0}^{+}(\textbf{r})=n_{0}^{+}(r_s) \Theta (R_\text{cl}-r)$, where $\Theta (x)$ denotes the Heaviside step-function and $n_{0}^{+}(r_s)$ is the constant bulk density, which depends only on the Wigner-Seitz radius $r_{s}$: ($4\pi r_{s}^{3}/3)=1/n_{0}$. The total number of electrons in the neutral clusters, $N_{e}$, is then given by $N_{e}=(R_{\text {cl}}/r_{s})^{3}$. Thus, the size of each closed-shell cluster is determined by the density parameters $r_s$ and the total number of electrons, $N_{e}=588$. According to the polymer’s ELFs (see Fig. 1), and using the relation $\omega _p^2=4\pi n_o$, the most important Wigner-Seitz radii vary from $r_s = 1.00$ to 5.00 au. The jellium spheres corresponding to this range have sizes varying from $R_{\text {cl}} = 8.38$ to 1.68 au.

Although there have been minor refinements in terms of accuracy, the approach adopted in this work reflects the methodology used in^43,48,50,78^, and as such, it will be briefly explained in this section. In this approach, the time evolution of electronic density incorporates, in a non-perturbative manner, the complete dynamic interaction between an external field and the medium. This computational framework has been used to analyze various issues in condensed matter systems, such as dynamic charge screening in metallic media^79^, energy loss of atomic particles in matter^43,48,80^, as well as many-body effects associated with hole screening in photoemission^78^.

A static density functional theory (DFT) calculation is performed to obtain the system’s ground state. The time evolution of the complete electronic density, $n(\textbf{r},t)$, in response to an external field (in this case, a proton), is conducted within the framework of real-time TDDFT in the Kohn–Sham regime (atomic units are used throughout unless specified otherwise):

1 $$\begin{aligned} i\frac{\partial \psi _{j}(\textbf{r},t)}{\partial t} ~=~\left\{ T+V_\mathrm{{eff}}([n],\textbf{r},t)\right\} \psi _{j}(\textbf{r},t), \end{aligned}$$

where $\psi _j(\textbf{r},t)$ are the Kohn-Sham orbitals and *T* is the kinetic energy operator. The Kohn-Sham effective potential, $V_{\text {eff}}([n],\textbf{r},t)$, is a function of the electronic density of the system: $n(\textbf{r},t)= \sum _{j\in occ.}{\left| \psi _{j}(\textbf{r},t)\right| ^{2}}$. The effective potential $V_{\text {eff}}=V_\mathrm{{ext}}^+(\textbf{r})+V_\mathrm{{H}}([n],\textbf{r},t)+V_\mathrm{{xc}}([n],\textbf{r},t)+V_\mathrm{{p}}(\textbf{r},t)$ is obtained as the sum of the external potential created by the positive background of the jellium sphere $V_{\text {ext}}^+(\textbf{r})$, the Hartree potential $V_{\text {H}}([n],\textbf{r},t)$, the exchange-correlation potential $V_{\text {xc}}([n],\textbf{r},t)$, and the potential representing the projectile $V_\mathrm{{p}}(\textbf{r},t)$, which is modeled as a bare Coulomb charge. $V_{\text {xc}}([n],\textbf{r},t)$ is treated within a standard adiabatic local density approximation (ALDA) approach. The numerical procedure is that employed in Refs.^43,79–81^, where additional details can be found.

The energy loss is calculated by integrating the time-dependent induced force over the proton:

2 $$\begin{aligned} E_\mathrm{{loss}}(v)=-v\int _{-\infty }^{+\infty } F_z (t)dt, \end{aligned}$$

where *v* is the (constant) velocity at which the proton traverses the jellium. Once the induced force on the proton is calculated, the average or effective stopping power is computed as the energy loss per unit path length, i.e.,

3 $$\begin{aligned} \left[ \frac{dE}{dz}(v)\right] _{\text {TDDFT}}=\frac{E_\mathrm{{loss}}(v)}{2R_{\text {cl}}}. \end{aligned}$$

Recently, an alternative non-linear method has been introduced to characterize the stopping power of light and heavy ions in materials^52^. This method incorporates the influence of non-free electron distributions within a theoretical model for stopping power calculations, such as real-time TDDFT. For a low energy proton ($v < v_F$), the Penn approach has been used recently in the transport cross section (TCS)^49^. This approach considers the combination of electron-gas responses characterized by inhomogeneous densities, similar to the approach outlined in the Penn method^53^.

### Real-time TDDFT-Penn approach

To achieve this goal, each free electron density is analyzed based on the material’s ELF at the optical limit, as follows^52^:

4 $$\begin{aligned} g(\omega _p) =\frac{2}{\pi \omega _p}\text {ELF}(\omega _p). \end{aligned}$$

The stopping power depends on the plasmon frequency $\omega _p$, a value determined by the individual electron gas contributions obtained from $r_s$; $\omega _p=\sqrt{3}r_s^{-3/2}$. Therefore, the stopping power is now calculated as follows^52^:

5 $$\begin{aligned} \left[ \frac{dE}{dz}(v)\right] _{\text {TDDFT-Penn}} = \int _0^{\infty }d\omega _p g(\omega _p)\left[ \frac{dE}{dz}(v,\omega _p)\right] _{\text {TDDFT}}. \end{aligned}$$

In the above equation, the term $\left[ dE/dz(v,\omega _p)\right] _{\text {TDDFT}}$ is calculated in the real-time TDDFT framework using Eq. (3). Because of that, we named the Eq. (5) as the real-time TDDFT-Penn approach.

## Data Availability

The datasets used and/or analysed during the current study available from the corresponding author on reasonable request.
